# Prediction of Toxicant-Specific Gene Expression Signatures after Chemotherapeutic Treatment of Breast Cell Lines

**DOI:** 10.1289/txg.7204

**Published:** 2004-09-14

**Authors:** Melissa A. Troester, Katherine A. Hoadley, Joel S. Parker, Charles M. Perou

**Affiliations:** ^1^Department of Pathology and Laboratory Medicine and; ^2^Curriculum in Genetics and Molecular Biology, University of North Carolina at Chapel Hill, Chapel Hill, North Carolina, USA; ^3^Constella Health Sciences, Durham, North Carolina, USA; ^4^Department of Genetics and Lineberger Comprehensive Cancer Center, University of North Carolina at Chapel Hill, Chapel Hill, North Carolina, USA

**Keywords:** breast cancer, class prediction, doxorubicin, etoposide, 5-fluorouracil, gene expression, microarrays

## Abstract

Global gene expression profiling has demonstrated that the predominant cellular response to a range of toxicants is a general stress response. This stereotyped environmental stress response commonly includes repression of protein synthesis and cell-cycle–regulated genes and induction of DNA damage and oxidative stress–responsive genes. Our laboratory recently characterized the general stress response of breast cell lines derived from basal-like and luminal epithelium after treatment with doxorubicin (DOX) or 5-fluorouracil (5FU) and showed that each cell type has a distinct response. However, we expected that some of the expression changes induced by DOX and 5FU would be unique to each compound and might reflect the underlying mechanisms of action of these agents. Therefore, we employed supervised analyses (significance analysis of microarrays) to identify genes that showed differential expression between DOX-treated and 5FU-treated cell lines. We then used cross-validation analyses and identified genes that afforded high predictive accuracy in classifying samples into the two treatment classes. To test whether these gene lists had good predictive accuracy in an independent data set, we treated our panel of cell lines with etoposide, a compound mechanistically similar to DOX. We demonstrated that using expression patterns of 100 genes we were able to obtain 100% predictive accuracy in classifying the etoposide samples as being more similar in expression to DOX-treated than to 5FU-treated samples. These analyses also showed that toxicant-specific gene expression patterns, similar to general stress responses, vary according to cell type.

A stereotyped environmental stress response to a wide range of stressors and toxicants was first demonstrated in yeast (Gasch et al. 2000) and has subsequently been observed in a variety of mammalian cell models (Heinloth et al. 2003; Morgan et al. 2002; Murray et al. 2004; Park et al. 2002; Sesto et al. 2002; Weigel et al. 2002). We have previously used DNA microarray experiments to characterize the transcriptional responses of four breast cell lines to the chemotherapeutics doxorubicin (DOX) and 5-fluorouracil (5FU); these cell lines included two human telomerase reverse transcriptase (hTERT–immortalized human mammary epithelial (HME) cell lines and two tumor-derived cell lines of luminal epithelial origin (MCF-7 and ZR-75-1). A general stress response was shown to predominate when these cells were treated with DOX and 5FU (Troester et al. 2004). All four cell lines repressed genes involved in cell growth and induced DNA-damage response and xenobiotic metabolism genes, but there were differences in the general stress responses depending upon the cell type of origin of the cell line.

The mechanisms of action of DOX and 5FU are distinct. DOX is a topoisomerase IIA (TOP2A) poison. TOP2A is a nuclear enzyme that transiently breaks and rejoins the phosphodiester backbone of both strands of the double helix. As such, it is vital for DNA replication, chromosome segregation, and maintenance of chromosome structure. In previous studies (Tewey et al. 1984), DOX formed a stable ternary complex with DNA and TOP2A, thereby inhibiting the normal function of the enzyme. The complexed enzyme is unable to religate DNA so complex formation increases DNA strand breaks. TOP2A is highly expressed during S-phase, but TOP2A poisoning causes cell-cycle arrest in G_2_-M. The commonly used chemotherapeutic 5FU has several known mechanisms of action that distinguish it from DOX. 5FU covalently binds to thymidylate synthase, preventing *de novo* production of thymidine. It also incorporates into DNA and RNA (Longley et al. 2003; Pizzorno et al. 2000). The importance of each of these 5FU-mediated disruptions in cellular metabolism varies across cell lines and patients, but current studies emphasize the role of thymidylate synthase inhibition (Banerjee et al. 2002; Longley et al. 2003; Peters et al. 2002). Thymidylate synthase is highly expressed during S-phase, and its inhibition is thought to cause cell-cycle arrest in S-phase.

Using microarrays, it is often possible to identify unique patterns associated with specific toxicants in addition to common patterns of response. We used our panel of treated breast cell lines (Troester et al. 2004) to identify toxicant-specific expression signatures for DOX and 5FU. Cell lines derived from breast basal-like and luminal epithelium exhibited distinct toxicant-specific patterns of response. Using two statistical methods for class prediction, we then identified sets of genes that distinguished DOX- and 5FU-treated cells and used these lists to predict the mechanism of etoposide (ETOP), a drug that is mechanistically similar to DOX.

## Materials and Methods

### Cells and Cell Culture Conditions

ME16C and HME-CC cells, two basal-like hTERT-immortalized HME cell lines described by Torester et al. (2004), were gifts from J.W. Shay at the University of Texas Southwestern Medical Center at Dallas (Dallas, TX) and C. Counter at Duke University Medical Center (Durham, NC), respectively. ME16C cells and HME-CC cells were maintained in mammary epithelial growth media (Cambrex Bio Science Walkersville Inc., Walkersville, MD). MCF-7 cells (a gift from F. Tamanoi, University of California at Los Angeles) and ZR-75-1 cells (American Type Culture Collection, Manassas, VA) were maintained in RPMI 1640 with l-glutamine (GIBCO, Carlsbad, CA) supplemented with 10% fetal bovine serum (Sigma Chemical Co., St. Louis, MO) and 50 U/mL penicillin and 50 U/mL streptomycin (GIBCO). All cell lines were tested for mycoplasma by the University of North Carolina at Chapel Hill Tissue Culture Facility before experiments were conducted and at regular intervals thereafter. Cells were maintained at 37°C and 5% carbon dioxide.

### Cytotoxicity Assay

A mitochondrial dye conversion assay (Cell Titer 96; Promega Corp., Madison, WI) was used to measure cell viability after treatment. This assay was conducted according to manufacturer’s instructions, with modification as follows. Briefly, 5,000 cells were seeded per well of a 96-well plate. Cells were allowed to adhere overnight, and then media were replaced with fresh media containing a range of drug doses (DOX, 0–1 μM; ETOP, 0–500 μM; 5FU, 0–10 mM). After 36 hr of drug treatment, 15 μL of tetrazolium dye solution were added, and cells were incubated for 1 hr at 37°C before adding stop solution. Dye conversion products were solubilized in a humidified chamber overnight, and absorbance was measured at 570 nm (minus background absorbance at 650 nm). The 50% inhibitory concentration (IC_50_) for 36 hr of treatment with each drug in each cell line was estimated using nonlinear regression (SAS Statistical Software, version 8; SAS Institute Inc., Cary, NC) as described previously (Troester et al. 2004).

### Collection of mRNA for Microarray Experiments

Cell lines were grown in 150-mm dishes to 70–80% confluence and then treated for 12, 24, or 36 hr with toxicant at the IC_50_ concentration. The cells were harvested by scraping, and cell lysates were enriched for mRNA using a Micro-FastTrack kit (Invitrogen Corp., Carlsbad, CA). The reference RNA was generated by harvesting mRNA from each cell line at 80% confluence and pooling four such harvests (i.e., four MCF-7 harvests were pooled and served as reference mRNA for all MCF-7 experiments).

### Microarray Experiments

To synthesize labeled cDNA, reverse transcription reactions were carried out using 3 μg of mRNA as described previously (Perou et al. 2000; Troester et al. 2004). Briefly, 5FU, DOX, ETOP, and vehicle controls were labeled with Cy5–dUTP, and the pooled cell line control was labeled with Cy3–dUTP. The Cy3- and Cy5-labeled samples were combined and hybridized overnight at 65°C to a custom oligonucleotide microarray created in the University of North Carolina at Chapel Hill Genomics Core Facility. Arrays were spotted with Compugen (Jamesburg, NJ) human oligos representing approximately 22,000 genes. Two replicate arrays for each sample were selected for subsequent analysis. All micro-array raw data tables are available at the UNC Microarray Database (https://genome.unc.edu/) and have been deposited in the Gene Expression Omnibus (http://www.ncbi.nlm.nih.gov/geo/) under accession number GSE1647 (submitted by C. Perou).

### Significance Analysis of Microarrays

Genes that were significantly up- or down-regulated were identified using Significance Analysis of Microarrays (SAM; Tusher et al. 2001). For the SAM analysis, data were excluded for genes that did not have mean intensity greater than twice the median background for both the red and green channel in at least 70% of the experiments. The log_2_ of the median red intensity over median green intensity was calculated for each gene. Missing data were imputed using the SAM Add-In for Excel (Microsoft Corp., Redmond, WA) plug-in with 100 permutations and *k*-nearest neighbors (KNN) with *k* = 10. For each cell line, 12-, 24-, and 36-hr DOX–treated arrays were coded as one class and were compared the 12-, 24-, and 36-hr 5FU–treated arrays using a two-class, unpaired SAM. Delta values were adjusted to obtain the largest gene list with a false discovery rate < 5%. The effects of adding media would be present in the signatures of both compounds and would not be identified as significantly associated with either toxicant. However, because DOX was solubilized in water and ETOP and 5FU were solubilized in dimethyl sulfoxide (DMSO), we also collected mRNA from each cell line treated with DMSO only for 12, 24, or 36 hr (data not shown). We compared these DMSO-treated samples with sham (media only) samples for these same time points using SAM. The lowest false discovery rate obtained was 15.3% (15 genes with 2.29 false significant), which shows that the toxicant-specific changes we detected are highly unlikely to reflect changes induced by vehicle.

### Class Prediction

The number of genes needed to distinguish DOX and 5FU samples were identified using 10-fold cross-validation (CV) analysis using Prediction Analysis of Microarrays (PAM) and a KNN classifier. The KNN metric uses the Euclidian distance to determine the similarity of a sample to its *k* nearest sample neighbors. To select genes for the KNN method, we used a gene selection method that was first described by Dudoit and Fridlyand (2002); the KNN genes were identified in the training set according to the ratio of between-group to within-group sums of squares (Dudoit and Fridlyand 2002). The *n* top-ranked genes were used for each round of CV. The size of the gene subset was increased for subsequent rounds of CV. The set of *n* top-ranked genes that gave the highest average prediction accuracy during CV was also determined and reported. Gene selection using PAM was completed as described previously (Tibshirani et al. 2002). Genes were selected that yielded the greatest predictive accuracy in classifying DOX versus 5FU using a 10-fold CV analysis.

For class prediction, we performed a 10-fold CV analysis to iteratively optimize the list of genes and to determine prediction accuracies. Each round of CV would begin by splitting the samples into a training set (90% of the samples) and a test set (10% left-out samples), with gene selection and training being performed on the 90% and then used to predict the status of the withheld 10%. This was repeated 10 times, each time using a different 10% subset and a different gene set. Our reported prediction accuracies are the average of these iterative cycles of prediction for the optimized model. The results of all CV analyses using PAM and KNN are presented in Supplemental Data, Tables 1–4 (http://ehp.niehs.nih.gov/txg/members/2004/7204/supplemental.pdf). To independently assess the validity of these gene lists, we used them to predict class for ETOP samples; this analysis is independent because the ETOP samples were not used to train the predictor. The prediction accuracy for the ETOP samples are shown in Tables 2 and 3. For the four-class model, two samples were misclassified with PAM (HME-CC 12 hr and ME16C 12 hr) and two samples were misclassified with KNN (MCF-7 12 hr and ZR-75-1 36 hr) yielding the reported prediction accuracies of 75%.

### Clustering of Toxicant-Specific Responses

**Figure 1:**
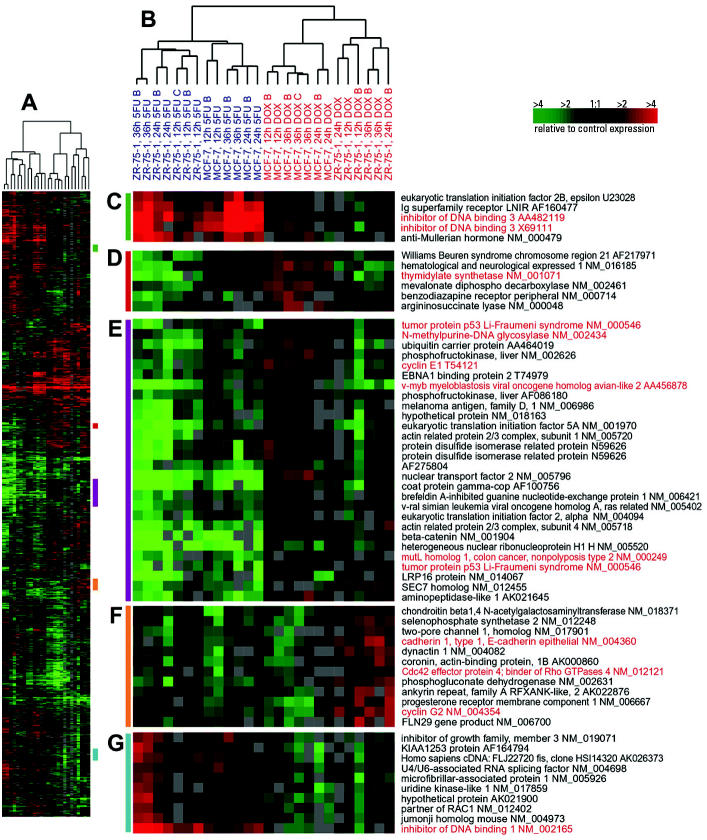
Gene expression patterns for genes that distinguish between DOX- and 5FU-treated luminal cells (MCF-7 and ZR-75-1). Hierarchical clustering analysis was conducted using 13 DOX-treated and 13 5FU-treated samples. Data from the union of the genes identified by SAM for MCF-7 and ZR-75-1 were identified and combined into a nonredundant list, and the compressed cluster is shown in *A* (complete cluster is available in Supplemental Data, Figure 1). Colored bars on right side of *A* illustrate the location of clusters shown in *C*–*G*. The dendrogram in *B* shows that the samples clustered into two groups according to treatment (DOX experiments labeled in red, 5FU experiments labeled in blue), but within each treatment branch, cell line–specific branches are also identifiable. Gene names and accession numbers are from Unigene (http://www.ncbi.nlm.nih.gov/entrez/query.fcgi?db=unigene). Gene names and accession numbers highlighted in red are discussed in text.

**Figure 2:**
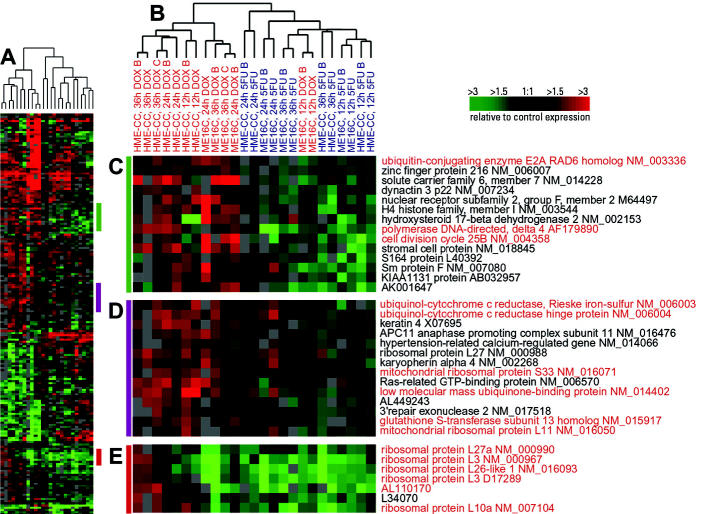
Gene expression patterns for genes that distinguish between DOX- and 5FU-treated basal-like cells (ME16C and HME-CC). Hierarchical clustering analysis was conducted using 13 DOX-treated and 12 5FU-treated samples. Data from the union of the genes identified by SAM for ME16C and HME-CC were identified and combined into a nonredundant list, and the compressed cluster is shown in *A* (complete cluster available in Supplemental Data, Figure 2). Colored bars in *A* illustrate the location of clusters shown in *C*–*E*. The dendrogram in *B* shows that the samples clustered into two groups according to treatment (DOX experiments labeled in red, 5FU experiments labeled in blue); however, there early time points for DOX-treated ME16C samples clustered with the 5FU-treated samples. Gene names and accession numbers are from Unigene (http://www.ncbi.nlm.nih.gov/entrez/query.fcgi?db=unigene). Gene names and accession numbers highlighted in red are discussed in text.

**Figure 3:**
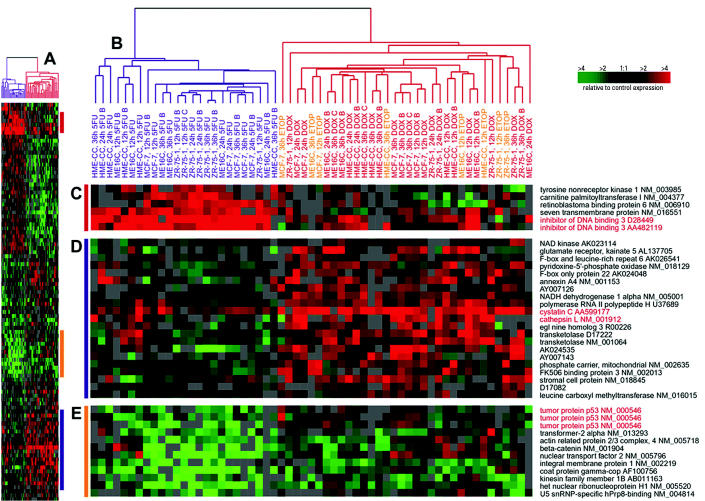
Gene expression patterns for genes selected for a two-class (DOX vs. 5FU) predictive model. Hierarchical clustering analysis was conducted using 26 DOX-treated, 25 5FU-treated samples, and 8 ETOP-treated samples. Data from the genes identified using a KNN classifier for DOX-treated versus 5FU-treated experiments are displayed in the compressed cluster shown in *A* (complete cluster available in Supplemental Data, Figure 3). Colored bars in *A* illustrate the location of clusters shown in *C*–*E*. The dendrogram in *B* shows that the samples clustered into two groups according to treatment (DOX experiments labeled in red, 5FU experiments labeled in blue and ETOP experiments labeled in orange. Gene names and accession numbers are from Unigene (http://www.ncbi.nlm.nih.gov/entrez/query.fcgi?db=unigene). Gene names and accession numbers highlighted in red are discussed in text.

Once gene lists were identified for the toxicant-specific responses of each cell line, hierarchical clustering analysis was conducted using the program Cluster (version 2.0; http://rana.lbl.gov/EisenSoftware.htm) to perform uncentered, average-linkage clustering; the data were visualized using Treeview (http://rana.lbl.gov/EisenSoftware.htm; Eisen and Brown 1999; Eisen et al. 1998). The gene lists generated with SAM for the luminal lines (MCF-7 and ZR-75-1) were combined into a nonredundant list, and data for these genes were compiled for all MCF-7 and ZR-75-1 samples. Similarly, the gene lists for the two basal-like lines (ME16C and HME-CC) were combined into a non-redundant list, and data for these genes were compiled for all HME-hTERT samples. For clustering and displaying results, data were excluded for genes that did not have mean intensity greater than twice the median background for both the red and green channels in at least 80% (Figures 1 and 2) or 70% (Figure 3) of the experiments. Supplemental Data, Figures 1–3 are (http://ehp.niehs.nih.gov/txg/members/2004/7204/supplemental.pdf) the complete cluster diagrams that correspond to Figures 1–3.

## Results

### Toxicant-Specific Transcriptional Responses

To investigate the toxicant-specific responses of four breast cell lines treated with chemotherapeutics, we collected mRNA from MCF-7, ZR-75-1, ME16C, and HME-CC cell lines after treating with DOX and 5FU at doses that produced similar levels of toxicity (IC_50_) across all four lines.

The IC_50_ was estimated from mitochondrial dye conversion assay results after 36 hr treatments with 5FU and DOX. The IC_50_ values and their 95% confidence intervals are shown in Table 1. For DOX and 5FU, the doses selected are consistent with physiologic doses expected in patients receiving treatment with DOX (Gewirtz 1999) or 5FU (Peters et al. 1993; Terret et al. 2000). This experimental design was aimed at defining the steady-state transcriptional response of these cell lines to toxicants and on defining chemotherapeutic-specific responses that were consistent over time. By combining 12-, 24-, and 36-hr–treated experiments into a single class for all supervised analyses, we identified genes that had a consistent pattern of expression across all three time points. These genes are the most likely to be consistent with *in vivo* experiments or patient samples, where it is difficult to assess how long a tissue sample has been exposed to a toxic agent. Although we did not specifically search for temporal variation in our SAM analyses, some temporal variation in gene expression can be observed in the clusters.

### Toxicant-Specific Responses in Luminal Cell Lines

A large list of genes was identified for MCF-7 (974 genes with 44.7 false significant) and for ZR-75-1 (883 genes with 41.6 false significant) when supervised analyses were conducted to compare DOX-versus 5FU-treated samples. Hierarchical clustering analysis of the MCF-7 and ZR-75-1 experiments using the combined and nonredundant gene lists showed distinct responses for each toxicant (Figure 1 and Supplemental Data, Figure 1). The primary dendrogram branches for DOX-treated and 5FU-treated experiments were subdivided into MCF-7 and ZR-75-1 branches (Figure 1B); this suggests that most variation in these genes is attributable to the toxicant, but that cell lines also contribute to the variation. A total of 191 genes (77 down-regulated and 114 up-regulated) appeared on the SAM lists for both MCF-7 and ZR-75-1. However, there are many more genes that show qualitative similarity in the toxicant-specific responses of MCF-7 and ZR-75-1 cells (Figure 1 and Supplemental Data, Figure 1) than are captured using the strict SAM analysis. Figure 1D shows a cluster of genes that is up-regulated in MCF-7 cells after DOX treatment but is down-regulated in ZR-75-1 cells after both treatments; thymidylate synthase (*TYMS*) is included in this cluster. Recent studies have shown that thymidylate synthase, the target of 5FU, binds *p53* mRNA and regulates the expression of *p53* at the translational level (Chu et al. 1999; Ju et al. 1999). This is relevant because *p53* expression is slightly induced by DOX in MCF-7 cells but not in ZR-75-1 cells or by 5FU treatment in either cell line (Figure 1E).

The gene set in Figure 1E also shows that several other genes had slightly higher expression in MCF-7 cells treated with DOX, and that these genes were typically repressed in ZR-75-1 cells. For example, the mismatch repair gene mutL homolog 1 (*MLH1*) was unchanged by DOX, and *N*-methylpurine-DNA glycosylase (*MPG*)*,* a base excision repair gene, was repressed by 5FU. Both DOX and 5FU can cause DNA damage, but differences in the profiles of damage induced by each compound may account for differently regulated repair enzymes. Cyclin E1 (*CCNE1*) was also slightly induced in DOX-treated MCF-7 cells, as has been shown in previous studies (Arooz et al. 2000). *CCNE1* and v*-myb* myeloblastosis viral oncogene homolog avian-like 2 (*MYBL2*) are important genes involved in the G_1_–S transition and are transcriptional targets of E2F (Yasui et al. 2003).

Figure 1F shows that ZR-75-1 cells have a unique response to DOX compared with MCF-7 cells and 5FU-treated cells. In concordance with increased E-cadherin (*CDH1*) expression shown in this cluster, an increase in (*CDH1*) mRNA (and CDH1–mediated cell–cell adhesion) has been shown previously in another breast cancer cell line after treatment with DOX (Yang et al. 1999). Cyclin G2 (*CCNG2*) was also induced in ZR-75-1 cells treated with DOX. This cyclin is inducible by DNA damage in a p53-independent manner (Bates et al. 1996).

Figure 1C and G shows clusters of genes that are induced by 5FU in both cell lines and either unchanged or only modestly changed in DOX-treated lines. For example, inhibitor of DNA binding 3 (*ID3*) (Figure 1C) and *ID1* (Figure 1G) were strongly induced only in the 5FU-treated samples. The Id proteins control cellular differentiation and cell-cycle progression by preventing transcription factors from binding DNA (Norton et al. 1998). These proteins target basic helix–loop–helix proteins that regulate cell-type–specific and cell-cycle–regulatory gene expression (Lassar et al. 1994); however, the role of these proteins in the response to 5FU is not known.

### Toxicant-Specific Responses in Basal-Like Cell Lines

A smaller list of toxicant-specific genes was identified for ME16C (76 genes with 3.7 false significant) and HME-CC (193 genes with 8.6 false significant) cells when SAM was used to compare DOX-treated with 5FU-treated samples. Hierarchical clustering using the combined and nonredundant gene lists for these two cell lines showed that there were distinct responses by toxicant (Figure 2 and Supplemental Data, Figure 2). However, the primary dendrogram branch for 5FU-treated basal-like cell lines also included two early time points for DOX-treated ME16C (Figure 2B). The 12-hr ME16C time point has many gene expression changes in response to treatment (Troester et al. 2004), but this time point does not exhibit the same toxicant-specific signature as do the 24- and 36-hr time points. These temporal differences likely account for the grouping of toxicant-specific signatures in Figure 2. As we have also seen in our previous study of the general stress response of these cell lines, the temporal response to these two toxicants varies by cell line.

Figure 2C shows a cluster of genes that is up-regulated in DOX-treated basal-like cell lines but down-regulated in 5FU-treated basal-like cells. These genes differ in both magnitude and direction of change. A number of these genes play a role in mediating DNA repair, including ubiquitin-conjugating enzyme E2A (*UBE2A*), which is a member of the RAD6 pathway that uses ubiquitin conjugation to control DNA damage–induced mutagenesis (Stelter and Ulrich 2003). Similarly, DNA polymerase delta is known to repair single-strand DNA interruptions produced during the process of base excision repair (Ho and Satoh 2003). Cell division cycle 25B (*CDC25B*), an important regulator of mitosis, is also found in this cluster.

The cluster in Figure 2D contains several mitochondrial genes (indicated in red). The altered expression of mitochondrial genes might be expected based on a recent study that demonstrated that anthracyclines, such as DOX, impair cellular respiration (Souid et al. 2003). Figure 2E consists of a set of genes that is clearly enriched for ribosomal proteins. Disruption of protein biosynthesis has been associated with alterations in the cell cycle and cell growth (Ruggero and Pandolfi 2003). Five ribosomal proteins are highlighted in red, and AL110170 is a hypothetical protein with 65% homology to ribosomal protein L22. The genes for these proteins are induced in the DOX-treated HME-CC cell line after 36 hr but are repressed in the ME16C cells at this and all other time points assayed.

### Class Prediction and Sample Classification for ETOP-Treated Samples

Having identified a number of genes that distinguish DOX- from 5FU-treated breast cell lines using SAM, we next performed class prediction analyses to assess whether these differences could be used to classify an independent data set collected using the same four cell lines. Because SAM does not perform sample classification, we used 10-fold CV with PAM (Tibshirani et al. 2002) and a KNN metric based upon the work of Dudoit and Fridlyand (2002). CV was implemented to optimize the number of neighbors (*k*) and the number of genes for KNN, and to optimize the shrinkage parameter (Δ) for PAM. Parameters were selected that generated the highest CV accuracy (internal validation) when distinguishing the DOX- and 5FU-treated samples. Then, using the optimized models, we made predictions on a test set of ETOP-treated samples (external validation). (Note that because CV excludes samples and the final model using the optimized parameters does not, the Δ-value selected during CV with PAM may correspond to a different number of genes during prediction. However, the number of genes selected in CV is held constant for the KNN-based prediction.)

We expected that because ETOP and DOX both inhibit TOP2A, their resulting transcriptional profiles should be similar. Therefore, we considered ETOP samples correctly classified if they were classified as DOX. In a two-class analysis (DOX vs. 5FU), we obtained a high degree of CV accuracy (80–98%) during training and a high degree of predictive accuracy (100%) in assigning the ETOP experiments as more similar to DOX than 5FU (Table 2). However, when we attempted to further subclassify the DOX and 5FU samples according to cell-type (basal-like–DOX vs. basal-like–5FU vs. luminal-DOX vs. luminal-5FU), our CV (76–80%) and prediction (75%) accuracies were diminished (Table 3). The errors in four-class prediction occurred in the 12-hr basal-like samples. This is not surprising based on our clustering results in Figure 2, where the early time points in one of the basal-like cell lines appeared distinct from later points.

To visualize the expression differences from the two-class DOX versus 5FU predictor using Euclidian KNN, we took these samples and the 100 gene set shown to be 98% accurate in prediction and performed hierarchical clustering analysis (Figure 3 and Supplemental Data, Figure 3). The similarities between the ETOP and DOX samples were observable across this gene set. This analysis showed two separate dendrogram branches in Figure 3B, with one branch containing all of the 5FU samples and the other containing the ETOP and DOX samples. Some of the genes identified in the earlier supervised analysis were recapitulated in this predictive gene set. Notably, *ID3* appears in Figure 3C and p53 appears in Figure 3E. An interesting cluster of genes that was more strongly induced in DOX and ETOP samples appears in Figure 3D, which includes cathepsin L (*CTSL*) and cystatin C (*CST3*). The activity of the cysteine protease CTSL is regulated by the cystatins (a family of cysteine proteinase inhibitors), and their imbalance is associated with increased invasiveness and development of the malignant cell phenotype (Kos and Lah 1998).

## Discussion

Most changes that occur in gene expression after treatment with either DOX or 5FU are indicative of a general stress response (Troester et al. 2004). However, in the work presented here, we were interested in identifying the toxicant-specific transcriptional responses to DOX and 5FU in breast epithelial cell lines. We conducted several different supervised analyses to identify genes that distinguished between DOX and 5FU and were able to define toxicant-specific profiles. Using SAM, we found that each cell type (basal-like or luminal-derived) and each cell line had unique responses to DOX and 5FU. Similar to our previous observations for general stress responses (Troester et al. 2004), we found that the luminal cell lines responded to treatment by regulating a large number of genes, whereas the basal-like cell lines had many fewer expression changes in response to treatment. In addition the basal-like cell lines showed greater temporal variation in expression than did the luminal lines. Some of the genes that comprised the general stress signature for each cell type were also found to have toxicant-specific expression in our supervised analyses. This occurred in cases where both DOX and 5FU induced or repressed gene expression relative to shams, but where one treatment induced a change with greater magnitude. For example, the expression of *CST3* was induced more strongly byTOP2A inhibitors than by 5FU (Figure 3D) but was induced in both treatments relative to sham (Troester et al. 2004). Thus, *CST3* is a general stress response gene with a toxicant-specific gene expression signature.

Toxicant-specific expression responses in our data were corroborated by published reports with these drugs in the same or similar cell lines. For example, impaired cellular respiration after DOX treatment has been previously reported (Souid et al. 2003), and in our data, mitochondrial gene expression was altered (Figure 2D). Earlier studies have shown that 5FU’s target protein thymidylate synthase can bind p53 (Chu et al. 1999; Ju et al. 1999), and we show that p53 mRNA levels are reduced in our 5FU-treated cells. Thus, many of the gene expression changes that we identified recapitulated previous findings. However, a number of significant changes that were not anticipated based on the literature were identified and likely have functional importance. For example, the induction of *ID1* and *ID3* has not previously been reported for 5FU. The importance of the Id proteins has only recently begun to be investigated (Norton et al. 1998); our findings suggest that these pathways may be responsive to toxicant treatment and warrant further investigation.

In addition to characterizing the toxicant-specific changes by cell line and cell type, we used toxicant-specific gene lists to make predictions on a third toxicant (ETOP) that is believed to have a similar mechanism of action as one of the training toxicants (DOX). Successfully classifying similar compounds establishes that observed transcriptional responses reflect an underlying mode of action. Using as few as 100 genes, we were able to classify ETOP samples as being similar to DOX treated samples with 100% predictive accuracy. This predictive accuracy was reduced to 75% when we attempted to further subclassify the DOX and 5FU samples according to cell type of origin. However, considering that with a four-class model, the likelihood of correctly classifying samples by chance is only 25% (compared with 50% for a two-class model), the four-class model still performs very well. The samples that were misclassified included the early time points in basal-like cell lines, which is consistent with our previous findings that the basal-like cell lines have a distinct expression profiles at 12 hr compared with their 24- and 36-hr time points (Troester et al. 2004).

We have used computational analyses to demonstrate that distinct transcriptional patterns can be identified for mechanistically dissimilar compounds and that toxicants with similar mechanisms can be classified accordingly. We selected two compounds with distinct mechanisms to train our model and a test compound with a mechanism similar to one of the training compounds. These kinds of mechanistic analyses are critical for predictive toxicology using gene arrays. Many studies in the field of toxicogenomics are aimed at populating databases with expression data for diverse toxicants with known mechanisms of action (Hamadeh et al. 2002). These databases can then be used to infer mechanism of action for new compounds. Our data show that this approach is feasible and identifies many new genes and pathways that are important in the response to these toxicants.

## Supplementary Material

Supplemental Figures and Tables

## Figures and Tables

**Table 1:** Estimated IC_50_ for 5FU, DOX, and ETOP based on mitochondrial dye conversion assay.^a,b^

	Cell line	IC_50_*^c^*	Treatment dose*^c^*
5FU	MCF-7	0.34 (0.13–0.55)	0.3
	ZR-75-1	3.3 (2.8–3.7)	3.0
	ME16C	0.064 (0.055–0.074)	0.06
	HME-CC	0.011 (0.009–0.013)	0.01
DOX	MCF-7	0.86 (0.74–0.97)	0.9
	ZR-75-1	0.43 (0.37–0.50)	0.4
	ME16C	0.52 (0.49–0.54)	0.5
	HME-CC	0.16 (0.14–0.18)	0.2
ETOP	MCF-7	35 (30–40)	40
	ZR-75-1	26 (8.6–43)	30
	ME16C	21 (18–23)	20
	HME-CC	6.1 (5.6–6.7)	10

a Values in parentheses represent 95% confidence intervals.

b Partially adapted from Troester et al. (2004); IC_50_ values for 5FU and DOX were previously reported.

c Doses for 5FU are in millimolar (mM); those for DOX and ETOP, micromolar (μM).

**Table 2:** Two-class CV and prediction accuracy for ETOP samples.

	CV accuracy		Prediction accuracy	
Method	PAM	KNN*^a^*	PAM	KNN*^a^*
No.	2,460 (2.75)*^b^*	100	279 (2.75)*^b^*	100
Accuracy	80%	98%	100%	100%

a *k* = 11.

b Δ-Value is shown in parentheses.

**Table 3:** Four-class CV and prediction accuracy for ETOP samples.

	CV accuracy		Prediction accuracy	
Method	PAM	KNN*^a^*	PAM	KNN*^a^*
No.	652 (3.5)*^b^*	100	465 (3.5)*^b^*	100
Accuracy	76%	80%	75%	75%

a *k* = 9.

b Δ-Value is shown in parentheses.
