# Supplementary material for: Prediction of Toxicant-Specific Gene Expression Signatures after Chemotherapeutic Treatment of Breast Cell Lines
Source: Environ Health Perspect. 2004 Sep 14;112(16):1607–13. doi: 10.1289/txg.7204 (PMC1247657; doi:10.1289/txg.7204)
Supplement: Supplemental Figures and Tables [file ehp0112-001607s1.pdf]

Supplemental Data, Figure 1. The complete hierarchical cluster diagram for the toxicant-specific gene expression patterns of luminal epithelium derived cell lines (MCF-7 and ZR-75-1).

Supplemental Data, Figure 2. The complete hierarchical cluster diagram for the toxicant-specific gene expression patterns of basal-like cell lines (ME16C and HME-CC).

Supplemental Data, Figure 3. The complete hierarchical cluster diagram for 100 genes selected using a k-nearest neighbors metric to classify samples by treatment. All four cell lines and three treatments (5FU, DOX, and ETOP) are represented.

Troester, M.A. et al.; Prediction of Toxicant-Specific Gene Expression Signatures Following Chemoterapeutic Treatment of Breast Cell Lines

Supplemental Data, Figure 1

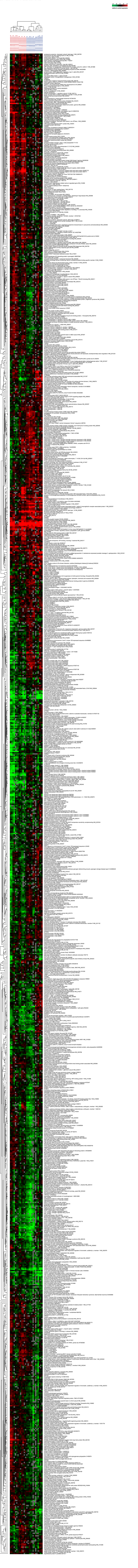

Supplemental Data, Figure 2

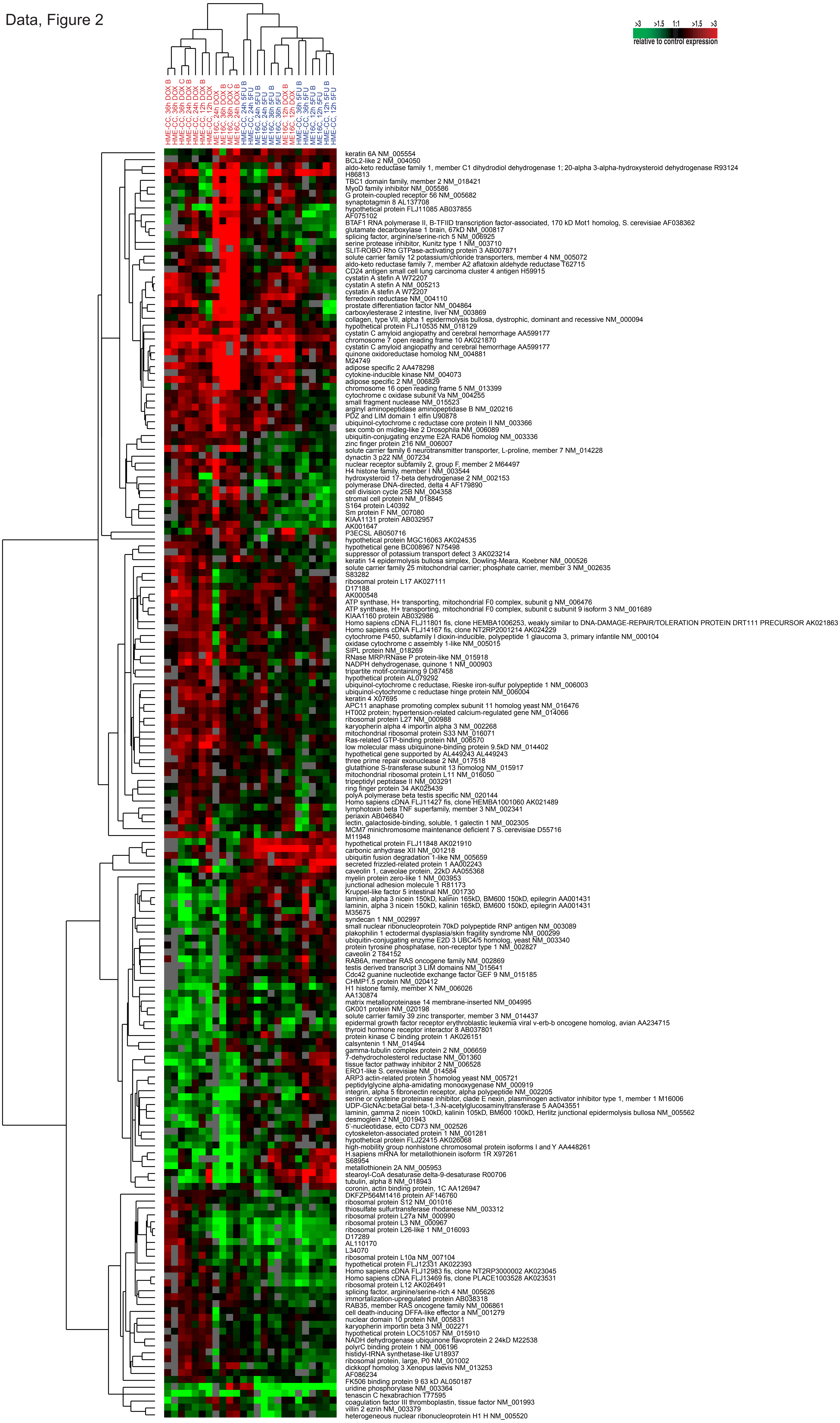

Troester, M.A. et al.; Prediction of Toxicant-Specific Gene Expression Signatures Following Chemoterapeutic Treatment of Breast Cell Lines

Supplemental Data, Figure 3

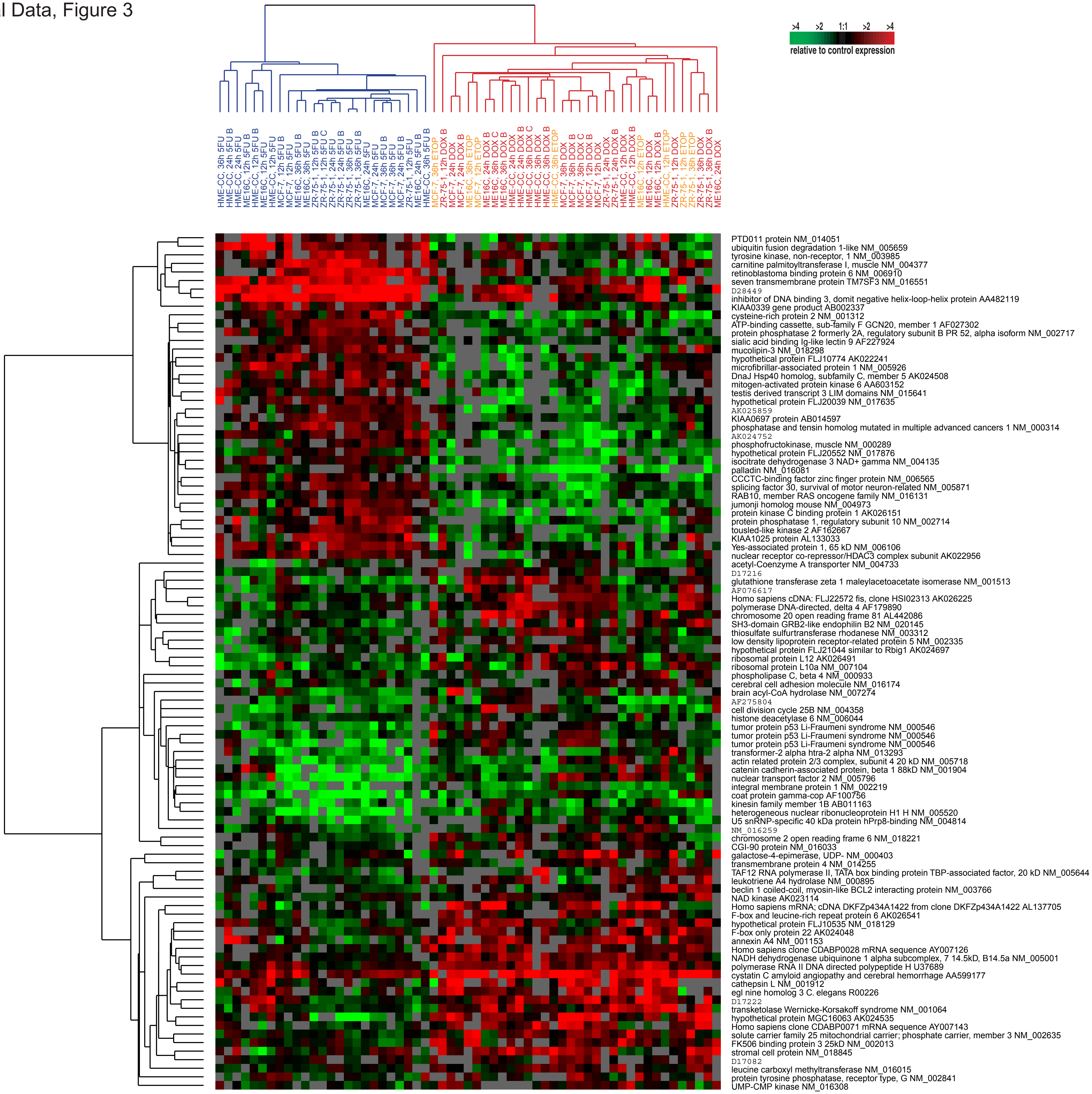

Table 1. Results of Cross-Validation (CV) Analyses Using 2-Class PAM Method

Delta Value	Average No. of Genes	Average CV Accuracy (%)
4	1123.1	64
3.5	1928.3	76
3	2323.6	78
2.75	2460.4	80
2.5	2584.6	76
2.25	2706.8	76
2	2829.4	76
1.75	2948	76
1.5	3075	76
1.25	3213.9	76
1	3371.1	74
0.75	3543	70
0.5	3716.2	70
0.25	3902.5	70
0	4077	70

Table 2. Cross-Validation (CV) Accuracy (%) Using 2-Class KNN Method

Number of Genes	k=	1	3	5	7	9	11
10		58	72	72	66	66	64
30		84	92	90	92	94	94
50		74	80	90	96	94	92
70		80	86	92	92	90	92
100		80	82	94	94	96	98
200		74	76	86	88	88	90
4077		66	64	70	68	68	76

Table 3. Results of Cross-Validation (CV) Analyses Using 4-Class PAM Method

Delta Value	Average No. of Genes	Average CV Accuracy (%)
6	17.4	66
5	45.6	68
4	136.9	76
3.5	652	76
3	1830.7	50
2.75	2211.2	46
2.5	2510.9	42
2.25	2763.2	48
2	2975.5	48
1.75	3183.5	48
1.5	3379.4	48
1.25	3549.1	46
1	3693.8	46
0.75	3826.3	46
0.5	3944.6	46
0.25	4037.7	42
0	4077	38

Table 4. Cross-Validation (CV) Accuracy (%) Using 4-Class KNN Method

Number of Genes	k=	1	3	5	7	9	11
10		40	50	48	54	56	56
30		60	62	76	76	72	76
50		66	78	74	70	70	74
70		62	74	76	76	78	68
100		58	66	70	70	80	78
200		54	58	64	62	76	74
4077		48	48	48	46	48	52
